# N-Linked Glycopeptide Identification Based on Open Mass Spectral Library Search

**DOI:** 10.1155/2018/1564136

**Published:** 2018-08-14

**Authors:** Zhiwu An, Qingbo Shu, Hao Lv, Lian Shu, Jifeng Wang, Fuquan Yang, Yan Fu

**Affiliations:** ^1^National Center for Mathematics and Interdisciplinary Sciences, Key Laboratory of Random Complex Structures and Data Science, Academy of Mathematics and Systems Science, Chinese Academy of Sciences, Beijing 100101, China; ^2^University of Chinese Academy of Sciences, Beijing 100049, China; ^3^Laboratory of Proteomics, Institute of Biophysics, Chinese Academy of Sciences, Beijing 100101, China; ^4^Computer Network Information Center, Chinese Academy of Sciences, Beijing 100101, China

## Abstract

Confident characterization of intact glycopeptides is a challenging task in mass spectrometry-based glycoproteomics due to microheterogeneity of glycosylation, complexity of glycans, and insufficient fragmentation of peptide bones. Open mass spectral library search is a promising computational approach to peptide identification, but its potential in the identification of glycopeptides has not been fully explored. Here we present pMatchGlyco, a new spectral library search tool for intact N-linked glycopeptide identification using high-energy collisional dissociation (HCD) tandem mass spectrometry (MS/MS) data. In pMatchGlyco, (1) MS/MS spectra of deglycopeptides are used to create spectral library, (2) MS/MS spectra of glycopeptides are matched to the spectra in library in an open (precursor tolerant) manner and the glycans are inferred, and (3) a false discovery rate is estimated for top-scored matches above a threshold. The efficiency and reliability of pMatchGlyco were demonstrated on a data set of mixture sample of six standard glycoproteins and a complex glycoprotein data set generated from human cancer cell line OVCAR3.

## 1. Introduction

Glycosylation plays a key role in nearly all biological processes such as glycose metabolism, signaling, cell adhesion, and cell proliferation [1]. Two main types of glycans have been reported: N-linked and O-linked glycans. N-linked glycans are attached to the Asn residue within a peptide sequence sequon of Asn-Xaa-Ser/Thr/Cys, where Xaa can be any amino acid except Pro. Furthermore, N-linked glycans own a five-monosaccharide core structure. The structures of O-linked glycans are more complex. We here focus on the analysis of N-linked glycopeptides.

Currently, high-throughput analysis of glycopeptides is typically achieved through tandem mass spectrometry combined with liquid chromatography separation (LC-MS/MS) [2]. However, compared with most of posttranslational modifications, glycans are large and heterogeneous and could be fragmented in MS/MS, resulting in difficulties in identifying glycopeptides. When collision-induced dissociation (CID) or high-energy collisional dissociation (HCD) is used to generate mass spectra of glycopeptides, the spectra are usually dominated by oxonium ions (fragment ions of the conjugated glycans) and glycopeptide related Y ions (peptide backbone ions carrying a glycan fragment from the glycosidic bond cleavage). According to our observation, more than 95% of glycopeptide spectra contain at least one of these ions, and the summed intensity of oxonium ions is over 50% of the total peak intensity in most of the spectra (data not shown). The intensities of b/y ions from peptide are lower than those of oxonium ions in general, and the absence of a part of b/y ions is quite common in the spectra of glycopeptides. Due to the relatively low abundance of glycopeptides compared to peptides without glycosylation, insufficient peptide fragment ions impede accurate identification of glycopeptides by LC-MS/MS. Various enrichment strategies and mass spectrometry-based workflows were thus developed and applied to both glycosylation site confirmation and intact glycopeptide identification [3–7]. The most popular strategy adopts assembly method. For example, some of existing software tools first identify peptide or glycan and then infer the other part based on the mass difference between the experimental precursor mass and the mass of identified peptide or glycan. The candidates for the other part can come from exiting database, e.g., glycan database of GlycomeDB [8], or identified peptide/glycan results of deglycopeptide spectra or product-dependent spectra. Such software tools include Byonic [9], protein prospector [10], GlycoPeptideSearch [11, 12], ArMone [13], GlycoFinder [14], GlypID 2.0 [15], GlycoFragWork [16], GlycoMaster DB [17], and pGlyco [18, 19]. This strategy, however, may lead to false inference of peptide or glycan with correct mass but incorrect sequence or structure. Moreover, these methods estimate the false discovery rate (FDR) separately for peptide and/or glycan identifications. For example, pGlyco estimates peptide FDR using the concatenated forward-reverse database search strategy and estimates glycan FDR using the spectrum-based decoy method followed by a finite mixture model to adjust the estimation bias. Finally, pGlyco assigns peptide and glycan identifications based on precursor mass and retention time to obtain final glycopeptide-spectrum-matches (GPSMs) [18]. However, to the best of our knowledge, there is still a lacks of widely accepted method for glycan FDR estimation.

Like peptide identification, existing software tools for glycopeptide identification are mostly based on sequence database search. Spectral library search, in which mass spectra are searched against previously identified spectra instead of protein sequences, is a promising alternative approach but has been less explored. However, few tools identify peptide and glycan together from a single glycopeptide spectrum except GPFinder [20] and MAGIC [21] as far as we know. GPQuest made the first attempt to identify glycopeptides using the spectral library search approach [22]. However, it still adopts the assembly strategy to infer glycan according to its mass calculated based on identified peptide and thus may lead to false inference of glycan. Moreover, it was written in Matlab, limiting its usability and running speed. Currently, the potential of spectral library for identification of glycan and peptide synchronously in a single HCD MS/MS spectrum has not been fully explored.

Here, we provide a novel spectral library search engine, named pMatchGlyco, for identification of N-linked glycopeptide from HCD spectra. As an extended version of pMatch [23], pMatchGlyco inherits multiple advantages of pMatch, such as library spectral optimization with peptide sequence information, powerful scoring system, and target-decoy strategy for quality control. To enable accurate glycopeptide identification, we extended the entire workflow of spectral library construction and searching. We first validated pMatchGlyco on a standard glycoprotein mixture data and finally identified more than 95.7% of high-confidence GPSMs given by pGlyco at 1% FDR. We then analyzed a glycopeptide data set generated from human cancer cell line OVCAR3 [24] and achieved 7,494 GPSMs identification results at 1% FDR, 64.3% more than GPQuest. In addition, we adopted two methods to validate our FDR estimation. pMatchGlyco was written in C++ and provides a graphical user interface, making it easy to use and running fast.

## 2. Materials and Methods

### 2.1. Materials

The spectra of six standard glycoproteins used in this study were described in Wen-Feng Zeng et al. [18]. Briefly, a mixture of six standard glycoproteins, including IgG, IgA, IgM, alpha-1-acid glycoprotein, alpha-2-macroglobulin, and haptoglobin, was first preprocessed through trypsinization and Hydrophilic Interaction Chromatography (HILIC) enrichment and then analyzed by nanospray LC-MS/MS on an Orbitrap Fusion Tribrid (Thermo Scientific). The experiment generated three types of spectra, i.e., HCD MS/MS, CID MS/MS, and HCD MS3 spectra. The first two types of spectra were generated from glycopeptides, and after MS/MS fragmentation, the peaks in certain m/z range of HCD MS/MS were selected for further fragmentation and the HCD MS3 spectra were collected. We used the HCD MS3 spectra to construct the library spectra to identify glycopeptides from HCD MS/MS spectra.

The MS/MS data set of human ovarian carcinoma cell line OVCAR3 was described in [24] and was downloaded from the PRIDE data repository (https://www.ebi.ac.uk/pride/archive/, data identifier PXD001571). Briefly, N-linked glycosite-containing peptide isolation was performed by the Solid Phase Extraction of Glycopeptide (SPEG) method (based on hydrazide chemistry), and the intact glycopeptides were enriched from OVCAR3 cells using a HILIC enrichment method. The tryptic peptides were analyzed by a Q-Exactive mass spectrometer (Thermo Fisher Scientific) using HCD. Here, the MS/MS data sets of ‘SunS_9_OVCAR3_Control_with_SILAC_SPEG_Deglycopeptides' and ‘SunS_9_OVCAR3_TMtreated_with_SILAC_SPEG_Deglycopeptides' were identified by pFind 2.8 [25, 26] to construct the library spectra to identify the glycopeptides from the MS/MS data set of ‘SunS_13_OVCAR3_HILIC_IntactGlycopeptide'.

### 2.2. Methods

**Figure 1:**
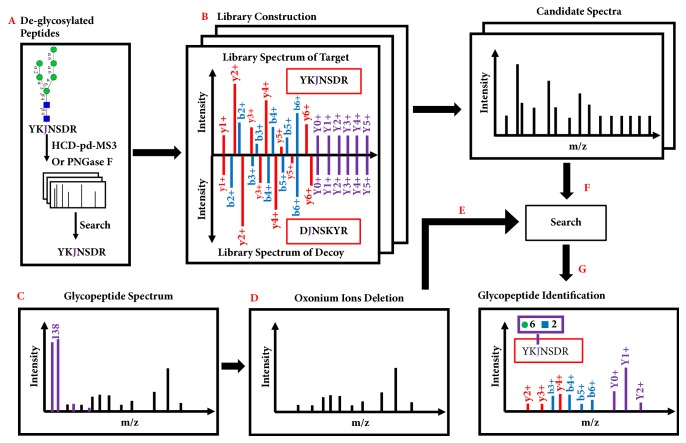
Workflow of pMatchGlyco. (A) Deglycosylated peptide identification through other search engines, e.g., pFind. The letter ‘J' represents Asn residue within a peptide sequence sequon of Asn-Xaa-Ser/Thr/Cys, where Xaa can be any amino acid except Pro. (B) Spectral library construction. Decoy spectra are generated for FDR estimation. (C) Glycopeptide spectrum with diagnostic peak with m/z of 138.054953 Da. The purple peaks represent oxonium ions. (D) Deletion of oxonium ions from the query spectrum in (C). (E-F) Selection of candidate library spectra for each glycopeptide spectrum. (G) Scoring the matches between query spectrum and candidate library spectra and reporting the best match.

pMatchGlyco supports a complete workflow of library search, including library construction, spectra matching and results evaluation (Figure 1). pMatchGlyco now only supports spectral data of ‘mgf' format. Users can convert raw data into this format using existing software tools, e.g., MSConvert utility of ProteoWizard [27] or pParse [28].

For OVCAR3 data set, raw data was converted to ‘mgf' format using pParse [28] with the* coeluted* parameter switched off. The deglycopeptide spectra were searched against RefSeq human protein databases [29] (downloaded from NCBI website July 29, 2013) by pFind 2.8 [25, 26]. The search parameters were set as follows: up to two missed cleavages were allowed for trypsin digestion, 10 ppm and 0.06 Da mass tolerances for precursor and fragment ions, respectively; carbamidomethylation (C) was set as a fixed modification and oxidation (M), deamidation (N), ‘Arg10' (R), and ‘Lys6' (K) were set as variable modifications; at most five modifications per peptide were considered for peptide identification.

#### 2.2.1. Library Construction

pMatchGlyco needs spectral identifications of deglycosylated peptides as input. The deglycosylated peptides can be obtained through techniques such as PNGase F cleavage [22] or HCD-product-dependent-MS3 (HCD-pd-MS3) [18], and then the corresponding spectra can be identified using common search engines such as pFind [25, 26]. pMatchGlyco constructs spectral library by combining spectra with the same identification results and optimizing the spectra with the peptide sequence information. As in pMatch [23], the common peaks in duplicate spectra are combined into a consensus peak with the averaged intensity value. For each consensus spectrum, a theoretical spectrum is generated with theoretical peaks with the uniform intensity value one. The relative intensities of the peaks in the theoretical and consensus spectra are multiplied by the factor of *θ*  (0 ≤ *θ* ≤ 1) and 1 − *θ*, respectively, and then the two spectra are merged by superimposing their common peaks. One difference from pMatch in constructing library is that pMatchGlyco uses the theoretical masses of precursor and annotated fragment ions instead of the average masses of experimental duplicate spectra. This is because the precursor and fragment mass tolerances of library spectra may be different from the query spectra. For example, the fragment mass tolerance of MS3 spectra in the data set of six standard glycoproteins is 0.5 Da, while the fragment mass tolerance is 20 ppm for query spectra. If we still use average masses, the correct library precursors and annotated fragment ions may fail to match. Most importantly, for pMatchGlyco, Y_0_~Y_5_ ions are added to each optimized spectrum with 40% relative intensities in order to match the Y ions in N-linked glycopeptide spectra. This step requires an additional parameter (modification_remove) for the modification type introduced by PNGase F (Deamidation on Asn) or HCD-pd-MS3 (HexNAc on Asn), and all the characters of N (Asn) in peptide sequence that are the N-linked glycosylation sites are changed to J.

#### 2.2.2. Spectral Matching

Given a query spectrum, pMatchGlyco first judges whether it was generated from a glycopeptide based on the diagnostic peak with m/z of 138.054953Th (internal fragment of HexNAc) [18]. Before matching, pMatchGlyco preprocesses the glycopeptide spectrum as follows. Isotopic peaks are removed and at most 6 peaks per 100Th are reserved for subsequent matching. In addition, a total of 25 types of oxonium ions are also removed [18, 21] (Supplementary Table S1). A library spectrum is selected for matching with the query spectrum if the precursor mass of the library spectrum plus the mass of one glycan in glycan database matches the precursor mass of query spectrum within a given tolerance. pMatchGlyco employs two subscores, i.e., a spectral dot-product score (DPS) and a probability-based score (PS), to find out the best identification for each query spectrum. The DPS is calculated to measure the similarity degree between a query and the *i*th candidate library spectrum in terms of matched peak intensity:

(1) $$\begin{array}{c} DPS=\frac{\sum_{Match\_peaks}I_{Q}I_{L}}{\sqrt{\sum_{Query\_peaks}I_{Q}^{2}}\sqrt{\sum_{Library\_peaks}I_{L}^{2}}} \end{array}$$

where* Match_peaks* denotes the set of matched peak pairs,* Query_peaks* and* Library_peaks* denote the set of all peaks in query and library spectrum, respectively, and *I*_*L*_ and *I*_*Q*_ denote the intensities of the library peak and the query peak, respectively.

PS is calculated to evaluate how the *i*th candidate library spectrum is outstanding from the others in terms of matched peak count:

(2) $$\begin{array}{c} PS=\sqrt{-log\left(\overset{n}{\underset{j=k_{i}}{\sum }}\left(\begin{array}{c} n\\ j \end{array}\right)P^{j}{\left(1-P\right)}^{n-j}\right)} \end{array}$$

where *n* is the number of peaks in the query spectrum with relative intensities no less than 0.05 and ranked in the top 12, and *k*_*i*_ denotes the number of annotated peaks in the *i*th library spectrum that are matched to the top *n* peaks in the query spectrum. *P* denotes the probability that each of the top-*n* query peaks matches by chance to the annotated peaks in the *i*th candidate spectrum, so $\sum_{j=k_{i}}^{n}\left(\begin{array}{c} n\\ j \end{array}\right)P^{j}{\left(1-P\right)}^{n-j}$ represents the probability that *k*_*i*_ or more peaks matched to the library spectrum by chance. It is quite like the p value in statistics. The larger *k*_*i*_ is, the smaller the probability is and hence the larger the PS is.

The *P* is calculated as

(3) $$\begin{array}{c} P=1-{\left(\;1-p\;\right)}^{m_{i}} \end{array}$$

where *m*_*i*_ denotes the number of annotated peaks in the *i*th library spectrum and *p* is the probability of random match between two arbitrary peaks and is estimated as

(4) $$\begin{array}{c} p=\frac{2\cdot pro\_tol}{mz\_range} \end{array}$$

where *pro*_*tol* is the tolerance of product ions in MS/MS, and *mz*_*range* is the m/z range of MS/MS and is set as 2,000 Da by default. In pMatch, *p* is set as (∑_*i*=1_^*T*^*k*_*i*_/*n*)/(∑_*i*=1_^*T*^*m*_*i*_), where *T* is the number of candidate library spectra for the query spectrum and may become inaccurate when the number of candidate library spectra is very small. In contrast, the estimation of *p* in pMatchGlyco is only based on product tolerance and hence is more robust.

The final score named ‘pMatchGlyco_Score' is the product of DPS and PS:

(5) $$\begin{array}{c} pMatchGlyco\_Score=DPS\times PS \end{array}$$

Given a query spectrum, the pair of a library spectrum and a glycan with the highest pMatchGlyco_Score is regarded as the final GPSM for this query spectrum. In this regard, if the highest pMatchGlyco_Score is zero, this GPSM will not be outputted.

#### 2.2.3. Control of FDR

pMatchGlyco uses the target-decoy strategy to evaluate the FDR of GPSMs. In the library construction step, for each optimized spectrum (target spectrum), a decoy spectrum is generated with the same precursor mass and charge status. The decoy sequence is obtained by reversing the target sequence but maintaining the sequon of N-linked glycan, by which means the glycopeptide feature is reserved [16]. Then, the corresponding decoy spectrum is born with explained peaks (b/y ions) moved to the new m/z positions determined by its reversed sequence, and other peaks including Y0~Y5 ions are unchanged. pMatchGlyco filters search results by their pMatchGlyco_Scores and estimates the FDR using the formula FDR = D/T, where D and T represent the numbers of matches to the decoy and target spectra [30], respectively.

## 3. Results

We first evaluated pMatchGlyco using an MS/MS data set produced from a mixture sample of six standard glycoproteins, which was used by pGlyco [18]. With 1% FDR at both peptide-spectrum-match (PSM) level and glycan-spectrum-match (GSM) level, 556 GPSMs were confidently identified using pGlyco and these results were further grouped into three subsets manually based on reliability level. The first subset contained 280 GPSMs that were confirmed by sufficient peptide fragment ions of their HCD MS/MS spectra and were selected as the high-confidence subset. The second and third subsets contained 87 and 189 GPSMs, respectively (Supplementary Table S2). According to the results given by pGlyco, mean glycan score of the first, second, and third subsets were 87.2, 54.9, and 47.3 respectively. As expected, the first subset, which was the high-confidence subset, had the highest mean glycan score. Based on its inherited algorithm, pGlyco only considers matching of Y fragment ions when scoring HCD/CID MS/MS spectra; therefore it is unable to evaluate matching of peptide fragment ions for each GPSM. However, its score still reflects confidence of GPSM to a certain extent.

**Figure 2:**
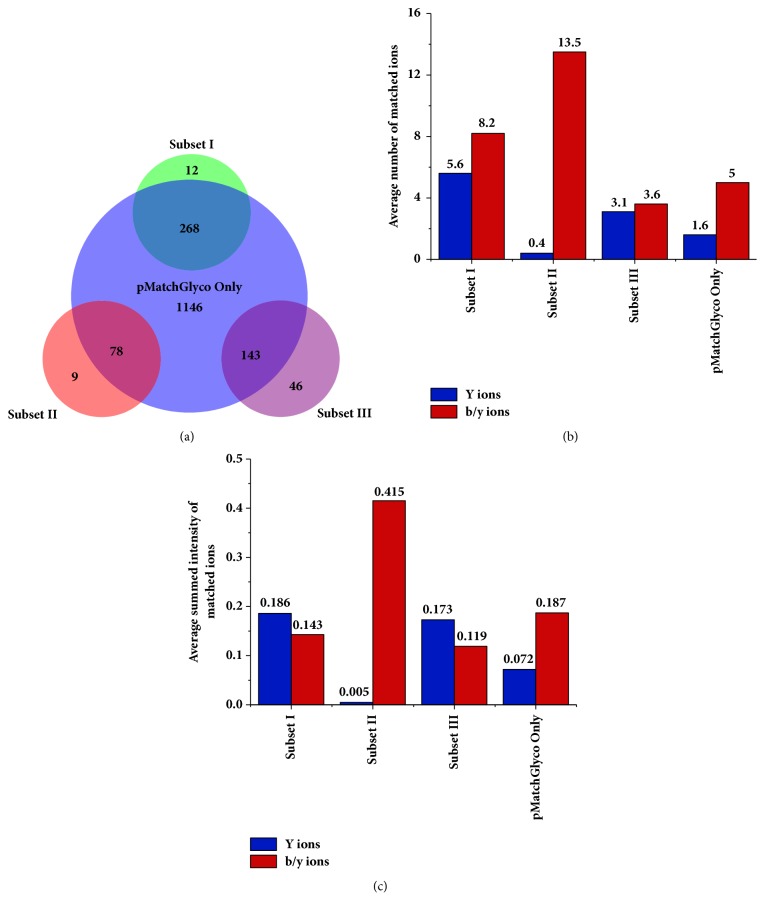
Comparison results of the data set of six standard glycoproteins between pGlyco and pMatchGlyco. (a) The overlap of the results. The results of pGlyco were grouped into three subsets manually based on reliability level. (b) The average numbers of matched ions for the four groups. (c) The average relative intensities of matched ions for the four groups.

The same data was reanalyzed using pMatchGlyco. We first built up the spectral library using the identification results of MS3 spectra searched by pFind 2.8.6 [25, 26] as described by Zeng et al. [18]. The identification results contained 409 glycosite-containing PSMs obtained at 1% FDR and resulted in 69 consensus library spectra after redundancy removal. Then, a total of 22,316 HCD MS/MS spectra from intact glycopeptides were searched against the spectral library. Finally, 1,635 target GPSMs were filtered out at 1% FDR (Figure 2; Supplementary Table S2). Among them, 268 (268/280, 95.7%) were from the high-confidence subset, and 267 of them shared the same glycopeptide identifications of pGlyco. The other one GPSM owned the identical peptide sequence but different glycan, and our result had a smaller mass error (1.35 ppm versus 4.71 ppm). This result suggested that pMatchGlyco could accurately identify high-quality glycopeptide spectra with a sensitivity of 96%. At the same time, there were 78 (78/87, 89.7%) and 143 (143/189, 75.7%) GPSMs from the second and third subsets, respectively, and 1,146 additional GPSMs obtained by pMatchGlyco (Figure 2, Supplementary Table S2).

Next, we checked the 12 GPSMs from the high-confidence subset for which pMatchGlyco could not output results at 1% FDR. Among them, 2 GPSMs owned the identical results but their scores were smaller than the threshold at 1% FDR, one GPSM had the identical peptide sequence but different glycan, and our result had a smaller mass error (2.57 ppm versus 7.87 ppm), 6 GPSMs had precursor mass errors more than 10 ppm (if we changed the precursor tolerance to 15 ppm, then these spectra could be identified with identical results with pGlyco at 1% FDR), and the other 3 GPSMs were matched to decoy results because no peaks in the corresponding target library spectrum matched the top 12 peaks in each query spectrum, which means no peaks can be picked to calculate PS score. Thus, their PS scores were zero in terms of target library spectrum match (Supplementary Table S2, Supplementary Figure S1).

We made comparison between the subsets I, II, and III and the “Other” set (those GPSMs identified by pMatchGlyco only) based on average number and summed relative intensity of matched peaks (Figures 2(b)-2(c)). We noted that the GPSMs in subset II showed quite small average number of matched Y ions (0.4). As we know, pGlyco used HCD/CID-MS/MS spectrum pairs to identify glycan, in which there were at least three trimannosyl core ions matched, or the (Y1, Y1*∗*) ion pair (Y1*∗* is peptide+cross-ring fragment of HexNAc [29]) with the same charge state was matched in the HCD MS/MS [18]. Therefore, we could infer that there must be enough Y ions in their corresponding CID MS/MS spectra, so it is unfair to compare the “Other” subset to subset II. We then compared the “Other” subset to the subset III and found that the average number and average summed relative intensity were smaller than those of subset III (1.6 versus 3.1) in terms of matched Y ions; they were larger in terms of matched b/y ions (5 versus 3.6). This indicated that the GPSMs from the ‘Other' set matched more peptide fragment ions than subset III and therefore they should be credible identifications. By the way, we also noted that the number of matched Y ions in ‘Other' subset was 1.6. If there were no three trimannosyl core ions matched in the corresponding CID spectra, these HCD spectra in ‘Other' subset would have no chance to be identified even though they had abundant peptide fragment ions.

We also used pMatchGlyco to analyze an MS/MS data set from real glycoprotein sample of human ovarian carcinoma cell line OVCAR3 [24]. For the deglycoprotein data set, 15,644 glycosite-containing PSMs were obtained at 1% FDR and resulted in 2,851 consensus library spectra after redundancy removal. The N-glycans with 85 different compositions identified by Sun [24] were used as glycan database.

**Figure 3:**
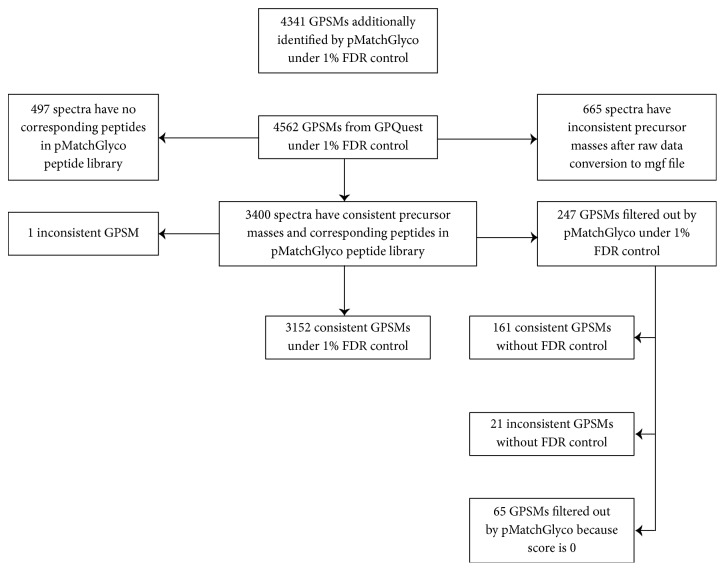
Comparison results of the OVCAR3 glycoprotein data set between the GPQuest and pMatchGlyco. From the 4,562 GPQuest results, we deleted 665 GPSMs because of inconsistent precursor masses after raw data conversion to mgf file and 497 GPSMs because of the lack of corresponding peptides in pMatchGlyco peptide library.

On the same data set, GPQuest identified 4,562 GPSMs at 1% FDR [24]. Among these GPSMs, we found that 1,162 spectra were theoretically unable to be consistently identified by two software tools, because (1) after raw data conversion to mgf by GPQuest workflow, 614 spectra had different precursor masses from those converted from pParse, which was used by pMatchGlyco workflow, (2) in the peptide library that pMatchGlyco built based on deglycosylated peptides, 497 GPSMs from GPQuest workflow did not have the corresponding peptides, and (3) 51 spectra had different precursor masses and meanwhile lacked corresponding peptides. Therefore the rest of 3,400 GPSMs, named gSet, were selected for a fair comparison with pMatchGlyco. At 1% FDR, pMatchGlyco identified 7,494 GPSMs, named pSet, of which 3,153 spectra (92.7%, 3,153/3,400) were in gSet. We found that only one spectrum in the 3,153 spectra (0.03%, 1/3,153) had different identification results between pMatchGlyco and GPQuest, indicating high consistency between the two software tools (Figure 3, Supplementary Table S3).

**Figure 4:**
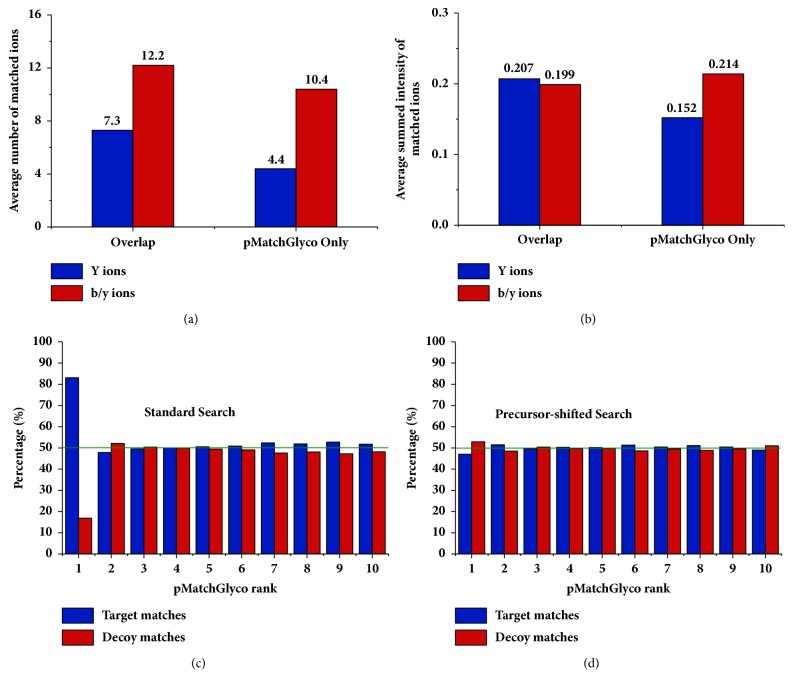
(a-b) Comparison between 3,153 consistent GPSMs and 4,341 additionally identified GPSMs by pMatchGlyco at 1% FDR in terms of average number and average summed intensity of matched ions. (c-d) Two methods to validate the FDR estimation method used by pMatchGlyco. All matches are shown and no filtering of any kind for output was performed. (c) The top 10 identifications from original spectra. (d) The top 10 identifications from the modified spectra in which each peak was shifted by 10 m/z unit except oxonium ions.

**Figure 5:**
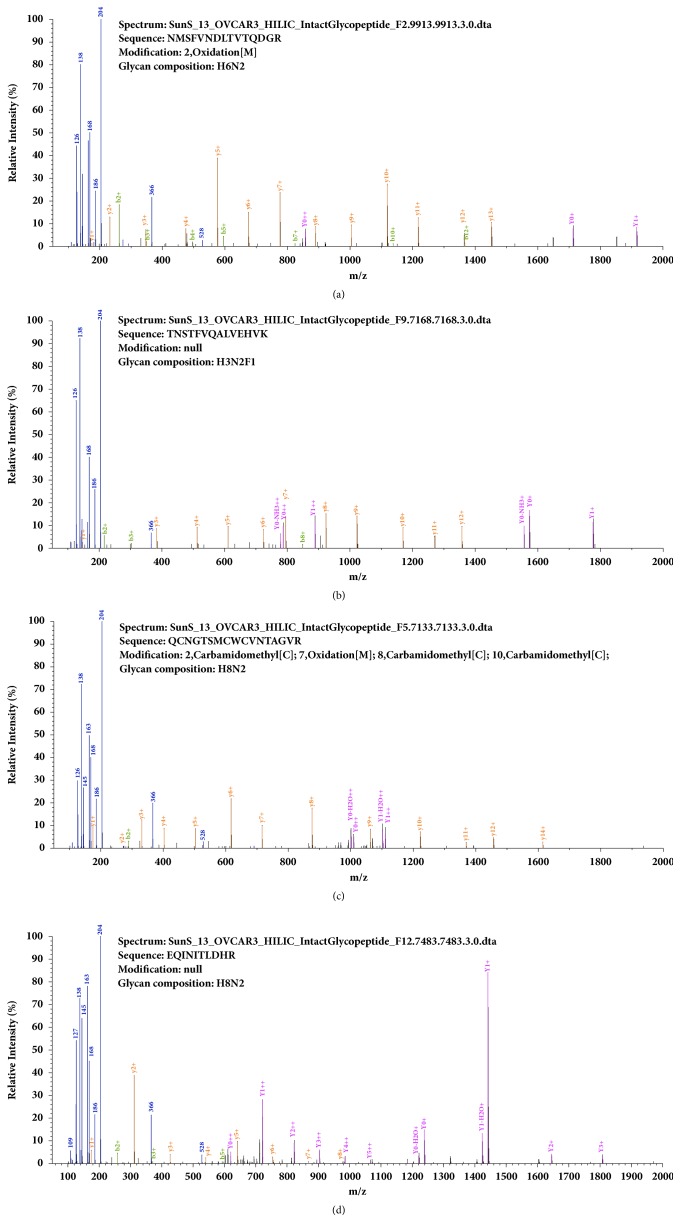
Four GPSMs results identified by pMatchGlyco only from the OVCAR3 data set. Oxonium ions were coloured in blue, peptide ions were coloured in orange (y ions) and green (b ions), and Y ions were coloured in purple. For glycan composition, H denotes hexose, N denotes N-acetylhexosamine, and F denotes fucose.

Among the other 247 (3,400-3,153) spectra, we found that 182 spectra (73.7%, 182/247) were also identified by pMatchGlyco but their scores were lower than the score threshold at 1% FDR, of which 161 GPSMs were identical with the results in gSet (65.2%, 161/247). In addition, the remaining 65 spectra (26.3%, 65/247) were not identified by pMatchGlyco because the top 12 peaks in the query spectra failed to match peaks from candidate library spectra, and their PS and pMatchGlyco_Score were 0 (Figure 3, Supplementary Table S3). Next, we compared the average number and summed relative intensity of matched peaks between the overlap set (3,153 GPSMs) and the unique part in pSet (4,341 GPSMs). Results showed that although the average number of matched peaks of 4,341 GPSMs was a bit smaller than the 3,153 GPSMs (14.8 versus 19.5), the average relative intensities of matched peaks were almost the same (0.366 versus 0.406), indicating that these 4,341 GPSMs were also credible identification (Figure 4). In Figure 5, four GPSMs that are only identified by pMatchGlyco prove that these GPSMs are reliable, because most of the intense peaks can be explained reasonably.

Finally, we adopted the two techniques proposed by Gygi et al. [31] on the OVCAR3 data set to further validate our FDR estimation method. First, we examined the pMatchGlyco identification results which ranked top 10 for all glycopeptide spectra. Rank 1 was highly enriched for target matches, whereas ranks 2-10 showed an equal percentage of target and decoy matches (Figure 4). Second, we created one data set of entirely falsified spectra by adding 10 m/z units to each peak (except oxonium ions) in every spectrum and then searched these spectra using pMatchGlyco. As expected, the GPSMs with the highest scores are distributed equally in target and decoy library spectra (Figure 4). These two facts coincided with the principle of target-decoy approach that incorrect matches have an equal chance of being derived from either the target or decoy database and thus justified our FDR estimation method.

## 4. Discussion

When applying pMatchGlyco to OVCAR3 data set, 665 spectra (14.6%, 665/4,562) showed inconsistent precursor masses between pParse output and the values given by GPQuest. In the results of GPQuest, the author pointed out that precursor masses of many spectra were corrected based on monoisotopic peak assignments. As shown in Supplementary Figure S2, coeluted precursor ions sometimes were overlapped in their isotopic peaks, which led to distorted isotopic peak distribution in terms of relative peak intensity and ambiguous monoisotopic peak assignment. This is not uncommon especially when a precursor ion has large mass and is multiply charged. The monoisotopic peak of a precursor ion is usually not the most intensive peak when its mass is large. But mass spectrometry will pick the most intensive peak within a mass isolation window for fragmentation and MS/MS analysis and record m/z of this chosen peak in the MS/MS scan. Commonly used data conversion tool, for example, MSConvert, will directly use the recorded precursor mass in MS/MS scan when converting raw data into mgf file, which leads to inconsistency between experimentally observed precursor mass and its theoretically monoisotopic peak. Another tool, RawConverter, tries to solve this problem [32]. We tested it and found that inconsistent precursor masses still existed. However, precursor mass will be consistent with its theoretically monoisotopic mass when isotopic peak distribution is normal, as shown in Supplementary Figure S3.

Another issue for OVCAR3 data set is lack of some deglycosylated peptides in our constructed peptide library, which explains 497 GPSMs given by GPQuest (10.9% 497/4,562). There were around one hundred peptides that cannot be found in peptide library after database search of deglycosylated peptide by pFind. We also searched the same data using Maxquant with the same parameters reported, whereas there were still ninety peptides that cannot be found. Furthermore, almost sixty peptides cannot be found in its own peptide set downloaded from web source. We therefore used pFind as our peptide search engine to keep consistency with our previous test data set from six standard glycoproteins.

One important feature of pMatchGlyco is that it adds Y_0_~Y_5_ ions into library spectra to match the corresponding ions in experimental glycopeptide spectra and considers the matched peaks in scoring. Compared to the method in which some user-specified Y ions are used as criterion to select candidate peptide or library spectra [22], our method is more automated and does not require users to have professional knowledge. This strategy is powerful to get the true peptides and glycans, which is demonstrated by the high consistency with pGlyco and GPQuest. However, it cannot distinguish glycans which are within precursor mass error. In this situation, pMatchGlyco simply selected the glycan with smaller precursor mass error. Results show that this method is efficient, and there was only one different GPSM in the overlap results both in six standard glycoproteins' data set and in OVCAR3 data set.

## 5. Conclusions

In this paper, we presented pMatchGlyco, an open spectral library search engine to identify N-linked glycopeptides from HCD MS/MS spectra. On the one hand, pMatchGlyco inherits the advantages of pMatch such as the optimization of library spectra using peptide sequence information and the well-designed scoring method. On the other hand, pMatchGlyco takes full consideration of the characteristics of N-linked glycopeptide spectra to improve sensitivity and accuracy. Moreover, in order to evaluate the FDR of GPSMs, pMatchGlyco also creates decoy library spectra by reversing the target sequence but maintains the sequon of N-linked glycan. Experiments showed that pMatchGlyco can efficiently identify intact N-linked glycopeptides and control their FDR well. On a data set from the mixture sample of six standard glycoproteins, pMatchGlyco identified more than 93.2% of high-confidence GPSMs given by pGlyco at 1% FDR. In a data set of complex sample of OVCAR3 from human cell line, 3,400 spectra had identical precursor masses after data conversion and 1% FDR filtering, and their corresponding deglycosylated peptides were identified at the same time by GPQuest and pMatchGlyco. From the 3,400 spectra, pMatchGlyco achieved a sensitivity of 92.7% (3,153/3,400). Furthermore, pMatchGlyco identified 4,341 more reliable GPSMs under 1% FDR. Last but not least, pMatchGlyco is easy to use and runs fast. We believe that pMatchGlyco will be a useful tool for the field of glycoproteomics.

## Data Availability

The MS/MS data set of six standard glycoproteins is available from the corresponding author upon request [18]. The MS/MS data set of OVCAR3 is deposited in the PRIDE repository (https://www.ebi.ac.uk/pride/archive/, data identifier PXD001571). The software tool of pMatchGlyco is freely available as a single compressed download that includes the main C++ executable, a user-friendly graphical user interface, example data files, and a complete user guide at http://fugroup.amss.ac.cn/software/pMatchGlyco/pMatchGlyco.html.

## Supplementary Materials

Supplementary 1 Supplementary Table S1: 25 types of oxonium ions.

Supplementary 2 Supplementary Table S2: pMatchGlyco and pGlyco identification results of the data set of six standard glycoproteins.

Supplementary 3 Supplementary Table S3: pMatchGlyco and GPQuest identification results of the data set of the OVCAR3 from human cell line.

Supplementary 4 Supplementary Figure S1: the match results for the three decoy GPSMs from the high-confidence subset of the six standard glycoprotein data pieces. Supplementary Figure S2: an example MS/MS scan of ambiguous monoisotopic peak assignment from OVCAR3 data set. The red arrow indicates monoisotopic peak of this precursor ion, and green arrow indicates the isotopic peak that was practically chosen for MS/MS, which is the third isotopic peak. This can be confirmed by m/z value included in the title of MS/MS scan, as blue arrow highlighted. Due to coisolation of another precursor ion, their isotopic peaks overlap. Interference of coisolated precursors distorted distribution of each cluster of isotopic peaks. Supplementary Figure S3: example MS/MS scans of ambiguous and unambiguous monoisotopic peak assignment from OVCAR3 data set. The red arrow indicates monoisotopic peak of this precursor ion, and green arrow indicates the isotopic peak that was practically chosen for MS/MS, which is the second (a, b, and c) or third (d, e) isotopic peak. This can be confirmed by m/z value included in the title of MS/MS scan, as blue arrow highlighted.
