# Supplementary material for: N-Linked Glycopeptide Identification Based on Open Mass Spectral Library Search
Source: Biomed Res Int. 2018 Aug 14;2018:1564136. doi: 10.1155/2018/1564136 (PMC6112209; doi:10.1155/2018/1564136)
Supplement: Supplementary 4 — Supplementary Figure S1: the match results for the three decoy GPSMs from the high-confidence subset of the six standard glycoprotein data pieces. Supplementary Figure S2: an example MS/MS scan of ambiguous monoisotopic peak assignment from OVCAR3 data set. The red arrow indicates monoisotopic peak of this precursor ion, and green arrow indicates the isotopic peak that was practically chosen for MS/MS, which is the third isotopic peak. This can be confirmed by m/z value included in the title of MS/MS scan, as blue arrow highlighted. Due to coisolation of another precursor ion, their isotopic peaks overlap. Interference of coisolated precursors distorted distribution of each cluster of isotopic peaks. Supplementary Figure S3: example MS/MS scans of ambiguous and unambiguous monoisotopic peak assignment from OVCAR3 data set. The red arrow indicates monoisotopic peak of this precursor ion, and green arrow indicates the isotopic peak that was practically chosen for MS/MS, which is the second (a, b, and c) or third (d, e) isotopic peak. This can be confirmed by m/z value included in the title of MS/MS scan, as blue arrow highlighted. [file 1564136.f4.docx]

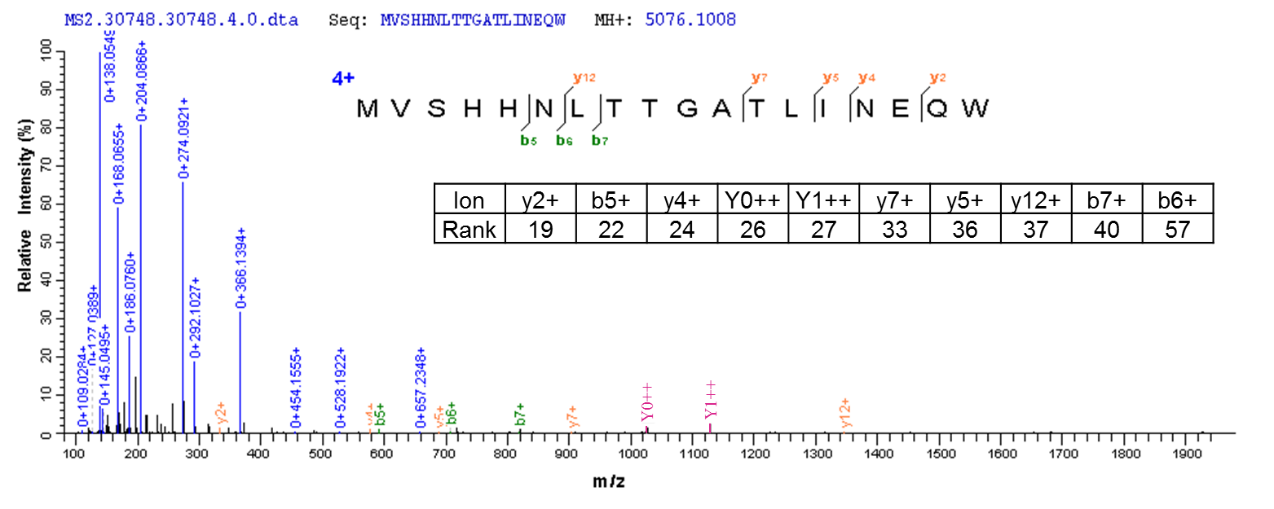


Matches in target spectrum


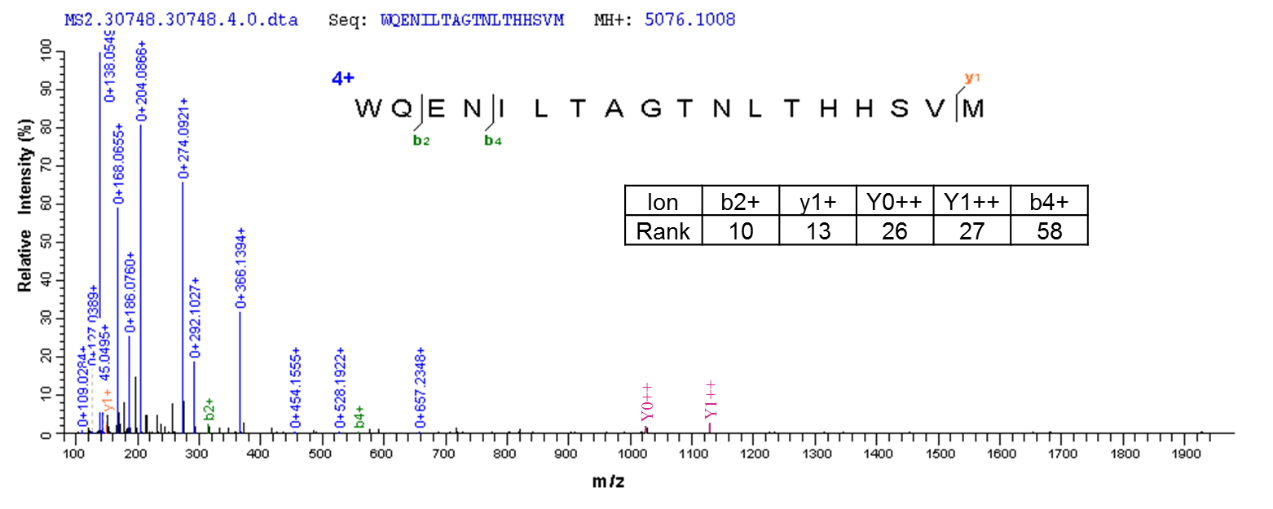

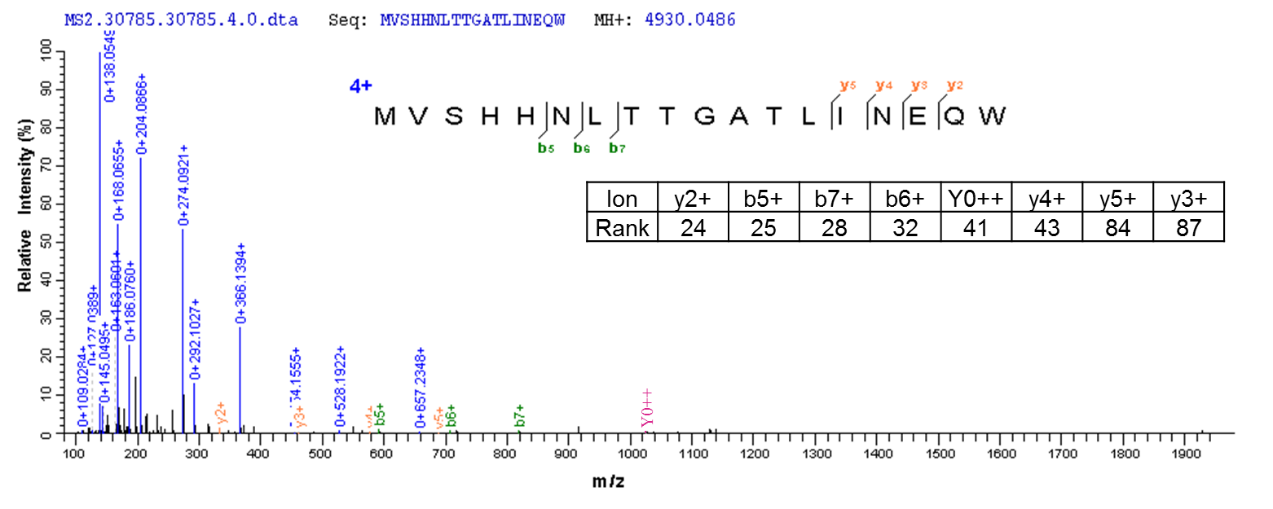


Matches in decoy spectrum

Matches in target spectrum


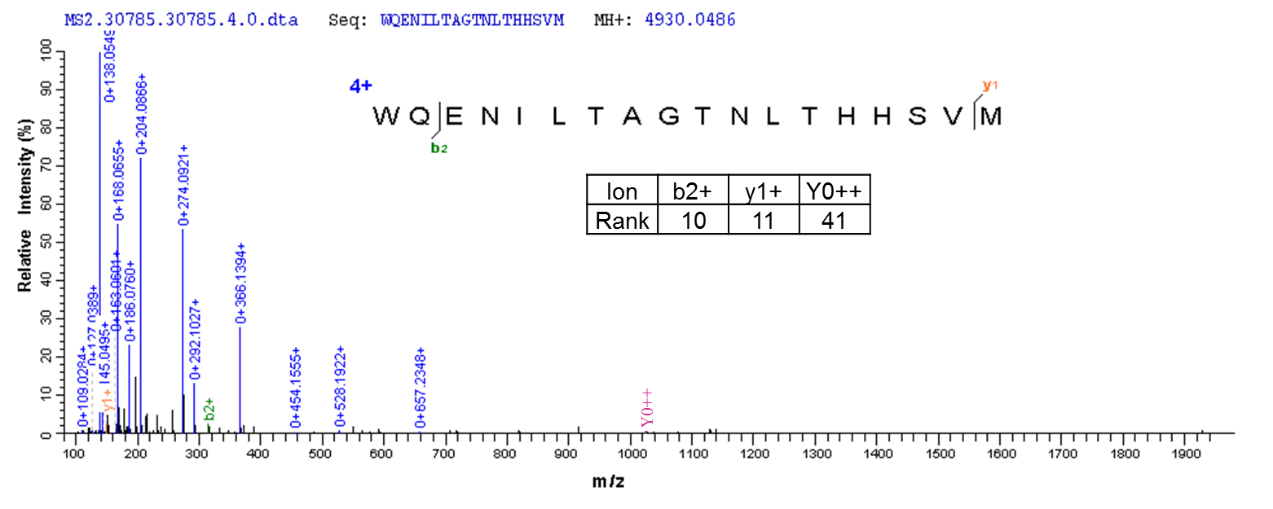


Matches in decoy spectrum


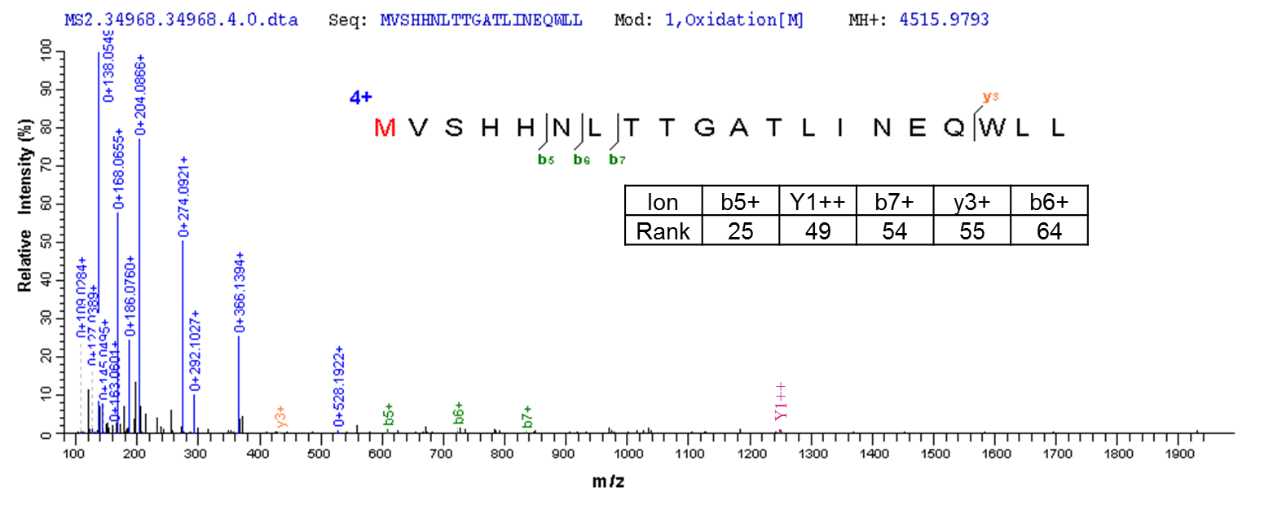


Matches in target spectrum


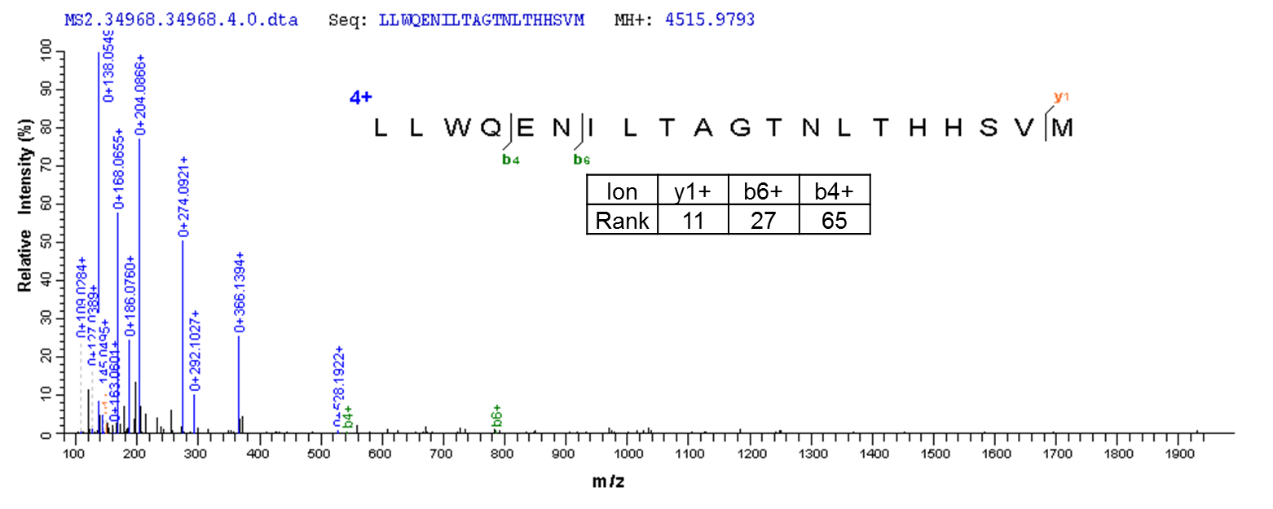


Matches in decoy spectrum

**Supplementary Figure S1.** Three decoy identifications given by pMatchGlyco from the high-confidence subset of the data set of six standard glycoproteins. This is because no peaks in the corresponding target library spectrum matched the top 12 peaks in each query spectrum, resulting in PS and pMatchGlyco_Score equal to zero.

**Supplementary Figure S2****.** Co-eluted precursor ions sometimes were overlapped in their isotopic peaks, which led to distorted isotopic peak distribution in terms of relative peak intensity, and ambiguous monoisotopic peak assignment.

**Supplementary Figure S3.** (a) An example MS/MS scan of unambiguous monoisotopic peak assignment from the OVCAR3 dataset. The red arrow indicates the monoisotopic peak of this precursor ion, and green arrow indicates the isotopic peak that was practically chosen for MS/MS, which is the second isotopic peak. This can be confirmed by m/z value included in the title of MS/MS scan, as the blue arrow highlighted.

**Supplementary Figure S3.** (b) An example MS/MS scan of unambiguous monoisotopic peak assignment from the OVCAR3 dataset. The red arrow indicates the monoisotopic peak of this precursor ion, and green arrow indicates the isotopic peak that was practically chosen for MS/MS, which is the second isotopic peak. This can be confirmed by m/z value included in the title of MS/MS scan, as the blue arrow highlighted.

**Supplementary Figure S3.** (c) An example MS/MS scan of unambiguous monoisotopic peak assignment from the OVCAR3 dataset. The red arrow indicates the monoisotopic peak of this precursor ion, and the green arrow indicates the isotopic peak that was practically chosen for MS/MS, which is the second isotopic peak. This can be confirmed by m/z value included in the title of MS/MS scan, as the blue arrow highlighted.

**Supplementary Figure S3.** (d) An example MS/MS scan of unambiguous monoisotopic peak assignment from the OVCAR3 dataset. The red arrow indicates monoisotopic peak of this precursor ion, and the green arrow indicates the isotopic peak that was practically chosen for MS/MS, which is the third isotopic peak. This can be confirmed by m/z value included in the title of MS/MS scan, as the blue arrow highlighted.

**Supplementary Figure S3.** (e) An example MS/MS scan of unambiguous monoisotopic peak assignment from OVCAR3 dataset. The red arrow indicates the monoisotopic peak of this precursor ion, and the green arrow indicates the isotopic peak that was practically chosen for MS/MS, which is the third isotopic peak. This can be confirmed by m/z value included in the title of MS/MS scan, as the blue arrow highlighted.
